# The influence of sex steroid hormones on the response to trauma and burn injury

**DOI:** 10.1186/s41038-017-0093-9

**Published:** 2017-09-14

**Authors:** K Al-Tarrah, N Moiemen, JM Lord

**Affiliations:** 1 Institute of Inflammation and Ageing, Birmingham University Medical School, B15 2TT, Birmingham, UK; 2 Scar Free Foundation Centre for Burns Research, University Hospital Birmingham Foundation Trust, B15 2WB, Birmingham, UK

**Keywords:** Burn, Trauma, Sex hormones, Sex steroid hormones, Testosterone, Estradiol, Oestrogen

## Abstract

Trauma and related sequelae result in disturbance of homeostatic mechanisms frequently leading to cellular dysfunction and ultimately organ and system failure. Regardless of the type and severity of injury, gender dimorphism in outcomes following trauma have been reported, with females having lower mortality than males, suggesting that sex steroid hormones (SSH) play an important role in the response of body systems to trauma. In addition, several clinical and experimental studies have demonstrated the effects of SSH on the clinical course and outcomes following injury. Animal studies have reported the ability of SSH to modulate immune, inflammatory, metabolic and organ responses following traumatic injury. This indicates that homeostatic mechanisms, via direct and indirect pathways, can be maintained by SSH at local and systemic levels and hence result in more favourable prognosis. Here, we discuss the role and mechanisms by which SSH modulates the response of the body to injury by maintaining various processes and organ functions. Such properties of sex hormones represent potential novel therapeutic strategies and further our understanding of current therapies used following injury such as oxandrolone in burn-injured patients.

## Background

There has been increasing interest in the role of the endocrine system in the pathophysiological response to major trauma, with several studies suggesting that sex steroid hormones (SSH) may influence the outcome after injury. Survival differences have been reported between males and females after major trauma, with female victims having lower mortality than males [1, 2]. Morbidity is also affected by gender and as early as 1975, McGowan et al. reported a significantly higher incidence of bacteraemic infections in male trauma patients compared to females, 58.5% vs 41.5% respectively [3]. In addition, adults show a gradual decrease in survival after traumatic injury with age [4]. This further suggests that the reduction in sex hormone, as a result of menopause and andropause, may influence the response to injury [5].

This review discusses the potential role of SSH in explaining gender and age differences in mortality and post-injury pathology and potential novel treatment strategies that target the endocrine response and may improve outcomes following trauma.

## Review

### Impact of gender on outcomes after trauma

Despite advances in medical care, sepsis and subsequent multiple organ failure (MOF) continue to be a major cause of morbidity and mortality in trauma patients [6], but there is evidence of gender differences for sepsis, MOF and mortality. In a study of 681,000 trauma patients, females demonstrated significantly lower complications and mortality rates compared to males [1]. A recent meta-analysis of 100,566 male and 39,762 female trauma patients found male gender was associated with higher incidence of complications, lengthier hospital stay and increased mortality [7]. In addition, male gender has been identified as a risk factor in the development of infection and MOF [8–10] and males suffer from significantly lower survival rates following sepsis when compared to females, 31% vs 74% [11]. This suggests that SSH may play a role in the maintenance of immune-inflammatory function in the trauma setting. This is further supported by the work of Haider et al. who concluded that females aged 13–64 exhibited significantly lower mortality outcomes following trauma-associated shock when compared to males and that this difference was abolished in the extremes of age when the effects of sex hormones were either absent or diminished [12]. Trentzsch et al., who performed a matched-pair analysis of 29,353 prospectively recorded trauma cases, concluded that males were more susceptible to MOF, sepsis and mortality [13].

Female patients appear to benefit from better physiological reserves and thereby are more protected against the consequences of trauma and shock. A prospective clinical study reported that female trauma patients required less fluid resuscitation volumes (12 L vs 8 L, *P* < 0.05), less Starling curve intervention (42% vs 15%, *P* < 0.05) to maintain oxygen delivery index and less inotrope and/or vasopressor support (36% vs 10%, *P* < 0.05) compared with similarly injured male patients and a standardized management protocol [14]. Another prospective clinical study involving more than 4000 patients reported that premenopausal women exhibited lower serum lactate levels and required less blood transfusion despite having more severe injuries [15].

However, the role of gender in modifying the response to trauma is still not clear-cut, with multiple conflicting clinical reports in the literature. Rappold et al. concluded that the female gender offered no protection from the development of acute respiratory distress syndrome, pneumonia or sepsis nor was it associated with decreased mortality rates post-trauma [16]. This finding was replicated by other studies which have demonstrated equivalent mortality rates in both genders following traumatic injury [17–19]. Other studies have suggested that female gender is a risk factor in trauma patients and is associated with increased complication and mortality rates [20–22]. These conflicting findings may be attributed to many factors such as study sample size, triage, treatment speed, and management protocol.

This apparent lack of agreement in the literature highlights the need for further studies in better controlled environments, comparing similar types of injury and taking age and gender into account in order to obtain more conclusive data. In addition, there remains a paucity of data on the mechanisms that may underlie gender differences in humans, with the majority of such research done in animal models of trauma. In this review, we therefore discuss the potential impact of gender and SSH on different aspects of the response to trauma, and we have made it clear where the data rely almost entirely on animal studies.

### Effect of SSH on the immune and inflammatory response to trauma

Various clinical and experimental studies have demonstrated that gender influences both humoral and cell-mediated immune responses and SSH receptors have been identified in multiple lymphoid tissues such as the bone marrow, spleen and thymus, as well as in different immune cells including lymphocytes, mast cells, granulocytes and macrophages [23]. Trauma has been shown to lead to immune dysfunction which, in turn, is associated with increased susceptibility to sepsis, MOF and mortality [24–27]. The processes driving immuneparesis after trauma are complex and include the cytokine storm elicited by tissue damage, which includes concomitant release of pro- and anti-inflammatory cytokines and the suppression of a variety of cell-mediated immune responses, which we have reviewed previously [5]. This immune suppression is mediated largely by the effects of cortisol released as a result of activation of the hypothalamic-pituitary-adrenal axis, but there is evidence that sex hormones represent an additional influence.

Wichmann et al. reported significant gender differences in B lymphocyte, T lymphocyte and natural killer (NK) cell counts following surgery despite comparable preoperative cell counts [28], with men showing reductions in cell numbers for up to 5 days. In addition, women exhibited a more pronounced pro-inflammatory response, with elevated circulating interleukin (IL)-6 levels, post-operatively [28]. Conversely, other studies have observed increased levels of IL-6, tumour necrosis factor-alpha (TNF-α) and procalcitonin in male trauma patients compared to females [29, 30]. What may be pertinent are the ratio of pro- to anti-inflammatory cytokines and the chronicity of the response: a profound initial inflammatory response may favour prevention of infection, but if inflammation is not resolved promptly, this can prevent wound healing and lead to organ damage.

Experimental studies in animal models of trauma have shown the modulation of immune responses by sex hormones. Overall testosterone appears to have anti-inflammatory and immunosuppressive effects, promoting synthesis of anti-inflammatory cytokines such as IL-10 by murine macrophages [31], reducing NK cell activity and the synthesis of pro-inflammatory cytokines, such as TNF-α, via the inhibition of nuclear factor kappa B (NFκB) [32, 33]. Testosterone has also been associated with decreased expression on macrophages and monocytes of toll-like receptor 4 (TLR4) which is involved in the activation of the innate immune system and production of inflammatory cytokines [34] by damage-associated molecular patterns (DAMPs).

Progesterone also exerts an immunosuppressive effect by inhibiting the activation of NFκB and increasing the expression of suppressor of cytokine signalling protein 1 (SOCS1) [35]. In addition, progesterone reduces the activity of macrophages and NK cells, as well as the synthesis of antibodies by B cells [36–39]. Elevated levels of progesterone during pregnancy have been associated with decreased development of pro-inflammatory helper T cell type 1 (Th1) immune responses while promoting the immune responses of Th2 including the synthesis of anti-inflammatory cytokines such as IL-4, IL-5 and IL-10 [40].

In contrast, estradiol has typically been shown to enhance cell-mediated and humoral immune responses. It augments NK cell cytotoxicity, as well as stimulating the production of pro-inflammatory cytokines including IL-1β, IL-6 and TNF-α [38, 41] and inhibits the synthesis of anti-inflammatory cytokines such as IL-10 [42]. In addition, oestrogens have been shown to increase survival and prevent apoptosis of immune cells [43, 44]. The balance of sex hormones in the circulation may thus be a key modulator of immune responses to trauma and tissue injury in humans.

Several murine studies have shown depressed immune responses in males as well as oophorectomized and aged females following trauma, haemorrhage and sepsis [45, 46]. Interestingly, pretreatment of female mice with 5-dihydrotestosterone (DHT) prior to trauma-haemorrhage resulted in depressed macrophage function and reduced levels of cytokines comparable to that seen in males [47, 48]. Moreover, castration and depletion of male sex hormones prior to trauma-haemorrhage resulted in enhanced immune responses [49–51]. In contrast, female sex hormones are associated with enhanced cell-mediated immune responses to trauma. Elevated systemic levels of estradiol in proestrus female mice played a pivotal role in post-trauma and haemorrhage immunocompetence [52]. Furthermore, administration of 17β-estradiol (E2) was associated with improved survival rates in animal models of sepsis [53]. A single dose of estradiol following trauma-haemorrhage and resuscitation was shown to restore depressed immune responses [54].

In animal studies, the effect of SSH on splenic immune response has been evaluated with studies demonstrating that E2 played a critical role in restoring splenic macrophage and immune functions post-injury by depressing pro-inflammatory cytokine production [52, 55]. Furthermore, Knoferl et al. reported that splenocyte proliferation and the release of IL-2, IL-3 and interf eron-gamma (IFN-γ) were suppressed in oophorectomised females following trauma-haemorrhage to levels comparable to those observed in males [52]. Moreover, castration prior to injury attenuated the depression of major histocompatibility complex (MHC) II (Ia) expression in mice, thereby improving cell-mediated immunity [56]. Oestrogen enhances splenic macrophage (TNF-α and IL-6) and T lymphocyte (IL-2 and IL-6) cytokine secretion following trauma [57–59]. In addition, E2 and estrogen receptor alpha (ER-α) agonist prevented the apoptosis of splenic dendritic cells and attenuated the depression of splenic dendritic cell cytokine production, co-stimulating factors and MHC II expression as well as antigen presentation capacities [60]. These effects of E2 on splenic function appear to be predominantly mediated via ER-α [59, 60]. This protective role of female sex hormones is associated with significantly increased survival rates in animal models [52].

Clinical studies investigating the effect of SSH on the immune-inflammatory cascade following trauma are more limited. Male patients of virtually all age groups have been reported to have higher incidence of sepsis following trauma and haemorrhagic shock suggesting the immunosuppressive effect of testosterone [61]. In addition, Zolin et al. reported that early elevations and increasing levels of testosterone over the initial 24-h period after injury were associated with an exaggerated inflammatory response and significantly increased risk of nosocomial infections and MOF. Interestingly, high circulating levels of estradiol at 24 h were associated with a fourfold greater risk of developing MOF [62]. Another study observed negative correlations between estradiol levels and TNF-α on day 1 and day 2 following trauma. However, no significant relationships were identified between SSH levels and IL-6, IL-8 or leukocyte counts [63]. Moreover, Lopez et al. concluded that while there is sexual dimorphism in the leukocyte genomic response following severe injury that are associated with more severe and prolonged organ failure, these differences were not in sex-linked genes or linked to differences in systemic levels of cytokines and therefore do not translate into sex-specific organ dysfunction or 28-day inhospital mortality [64].

The overall picture in relation to the impact of gender of the immune-inflammatory response to trauma and potential impact on outcomes such as sepsis, is one of a protective immune-enhancing role of oestrogens and a contrasting immunosuppressive effect of androgens. However, most data are derived from animal studies with very few studies in humans, and there is thus a need for clinical research and RCTs to determine benefits of SSH in maintaining immune competence after trauma.

### Effects of SSH on body systems after trauma

The actions of androgens, oestrogens and progestins are mediated through genomic and non-genomic pathways. The widespread expression of SSH receptors in tissues means that they have very broad effects on tissue and organ function, which may explain gender differences in trauma outcomes such as MOF (Table 1).

**Table 1:** Summary of the effects of oestrogen and testosterone on various organs

Organ	Oestrogen		Testosterone	
	Effect	References	Effect	References
Heart	• Improved left ventricular function • Improved cardiac output • Enhanced p38MAPK, Akt, eNOS and HSP expression • Reduction in IL-6, NFκB and TNF-α	[70–79]	• Depressed myocardial function • Suppression of Akt anti-apoptotic pathways • Reduced expression of Bcl-2 • Chronic administration improves function and reduces tissue damage	[80–82]
Lungs	• Decreased lung congestion, oedema and inflammation • Decreased emphysematous changes • Enhanced eNOS/PKG expression • Decreased KDC, MIF, TLR-4 and ERK expression • Reduction in IL-6, TNF-α, ICAM-1, CINC-1 and MIP-2	[87, 105–110, 159]	• Increased lung permeability and inflammation • Increased nitric oxide levels	[45]
Liver	• Reduction in liver congestion, portal inflammation and focal necrosis • Enhanced Kupffer cell function • Reduction in IL-6, TNF-α, MIP-1α and MIP-2 • Increased expression of Bcl-2 • Reduced ET-1 response	[68, 71, 87–96]	• Reduced hepatic microvascular blood flow • Diminished hepatocellular function	[97]
Spleen	• Stimulation of splenocyte proliferation • Increased IL-2 and IL-3 • Improved splenic macrophage and T lymphocyte function • Prevented apoptosis of splenic dendritic cells • Improved splenic dendritic cell function • Enhanced MHC II expression	[52, 55–60]	• Reduces MHC II expression • Depressed cell-mediated immune response	[56]
Intestines	• Reduced ET-1 response • Enhanced p38MAPK and Akt expression • Reduction in MPO, ICAM-1, CINC-1, CINC-3, MIP-2 and IL-6	[101–104]	• Enhances local pro-inflammatory response	[45, 97, 98]
Brain/Nerves	• Reduced iNOS expression • Reduction in hypothalamic TNF-α • Preservation of blood brain barrier integrity • Inhibition of MMP-2 and MMP-9	[113–117]	• Inhibition of caspase-3, MPO and XO activity • Reduction in malondialdehyde • Increased catalase levels • Maintains cellular and structural integrity • Preserves neural function	[118–120]
Kidneys	• Enhanced Akt and eNOS expression • Reduction in neutrophil infiltration	[127, 128]	• Reduced NOS, Akt and ERK expression • Low doses: increased IL-10 and reduction in T cell infiltration	[125, 126]

*MAPK* Mitogen-activated Protein Kinase; *eNOS* endothelial Nitric Oxide Synthase; *HSP* Heat Shock Protein; *IL* Interleukin; *NFκB* Nuclear Factor Kappa B; *TNF-α* Tumour Necrosis Factor-alpha; *PKG* Protein Kinase G; *KDC* Keratinocyte-derived Chemokines; *MIF* Migration Inhibitory Factor; *TLR* Toll-like Receptor; *ICAM* Intracellular Adhesion Molecule; *CINC* Cytokine-induced Neutrophil Chemoattractant; *MIP*; Macrophage Inflammatory Protein; *Bcl-2* B-cell lymphoma-2; *ET* Endothelin; *MHC* Major Histocompatibility Complex; *MPO* Myeloperoxidase; *iNOS* inducible Nitric Oxide Synthase; *MMP* Matrix Metalloproteinase

#### Cardiovascular system

Trauma and haemorrhage are known to induce myocardial dysfunction, decreasing cardiac output and blood flow [65, 66]. This effect is exacerbated in male mice, and castration 2 weeks prior to trauma and haemorrhage attenuates the depression of myocardial function [67]. Furthermore, treatment of male mice subjected to trauma and blood loss with an androgen receptor antagonist resulted in improved cardiovascular function [68]. In contrast, proestrus females have shown better regulation of cardiac function and blood volumes following trauma-haemorrhage when compared to males, with significant improvements in cardiac output and performance as well as increased circulating blood volume [69]. This effect may explain the improved restoration of organ function seen in proestrus female mice subjected to such injury [70].

In rodent studies, administration of E2 following trauma and haemorrhage significantly improved left ventricular function and cardiac output and prevented the increase of plasma IL-6 levels [71]. Furthermore, oestrogen has been shown to decrease IL-6 and NFκB in cardiomyocytes post-injury via inhibiting the expression and activity of hypoxia-inducible factor (HIF)-1α, resulting in improved cardiac function [72]. This inverse correlation between cardiomyocyte IL-6 levels and cardiac function was also reported by Yang et al. [73]. In addition, administration of E2 following trauma and haemorrhage increased the expression and activity of heme oxygenase (HO)-1 [74], mediated via the p38 mitogen-activated protein kinase (MAPK) pathway and subsequent phosphorylation of HSP-27 and αβ-crystallin [75]. Heat shock proteim (HSP)-27 and αβ-crystallin are known to prevent apoptosis during periods of stress, and Kan et al. showed that p38MAPK activation exerted further tissue protective effects through the increased expression and phosphorylation of endothelial NO synthase (eNOS) [76]. Furthermore, the cardioprotective properties of HO-1 post-oestrogen administration can also be achieved through Akt phosphorylation [77], which is also associated with inhibition of cardiomyocyte apoptosis [78, 79].

Testosterone has demonstrated both protective and detrimental cardiac effects following ischemic reperfusion insult in rodents. Acute testosterone replacement had adverse effects on myocardial function following injury, thought to be secondary to the inhibition of signal transducers and activators of transcription 3 (STAT-3) and suppression of cytokine signalling 3 (SOCS-3) anti-apoptotic pathways, as well as downregulation of Akt anti-apoptotic pathways that results in depressed myocardial function [80, 81]. In contrast, chronic testosterone therapy at physiological doses was effective at reducing infarct size, improving cardiac contractility, reducing arrhythmias, and improving myocyte viability as well as enhancing autonomic myocardial regulation following injury [82].

In addition to their effects on the myocardium, SSH has also been reported to modulate coagulation within the vasculature. A plethora of studies have implicated hormone replacement therapy and hormone-based contraceptives with promoting coagulation [83, 84]. Interestingly, female trauma patients were reported to be hypercoagulable on day 1 following injury compared to male trauma patients [85]. Furthermore, Gee et al. reported that early circulating estradiol-progesterone ratio levels positively correlated with thromboelastographic parameters and partial thromboplastin times and hence favouring a hypercoagulable state [63]. This early hypercoagulable state may in part explain why females presenting with acute traumatic coagulopathy following injury have been reported to have significantly poorer outcomes and twofold higher independent risk of mortality [86].

The influence of SSH on the human cardiovascular system following trauma is still poorly understood. Even though the observations from animal models appear promising, large-scale observational studies investigating various cardiovascular and hormone parameters of both genders as an initial step may prove insightful about the role of SSH on the human response to injury.

#### Gastrointestinal system

Following sepsis, female rats show less systemic endotoxemia and liver tissue damage than males and treatment with oestrogen and progesterone reduced liver congestion, portal inflammation and focal necrosis [87]. Administration of estradiol or androgen antagonist flutamide was associated with improved hepatocellular function following shock [68, 71]. This may be partly explained by the effect E2 exerts on Kupffer cells (KC). KCs are a major source of pro-inflammatory cytokines in the liver. Estradiol has been reported to enhance KC phagocytic capacity and depress cytokine production including IL-6, TNF-α, macrophage inflammatory protein (MIP)-1α and MIP-2 [88–91]. This is achieved via downregulation of TLR4-dependent p38MAPK and NFκB phosphorylation, while stimulating Akt activation and enhancing HO-1 expression [90, 92–94]. These beneficial effects of E2 on KC functions are mediated predominantly through ER-α [95], though a role for G protein-coupled receptor (GPR)-30, which activates protein kinase A (PKA) and increases expression of anti-apoptotic protein B-cell lymphoma-2 (Bcl-2) has been shown [96]. In contrast, testosterone had a deleterious effect following gut injury by producing pro-inflammatory and tissue toxic effects in mesenteric lymph nodes [97]. This negative impact of androgens is further supported by studies demonstrating that testosterone depletion ameliorated the magnitude of gut injury in animal models [45, 97, 98].

Maintenance of organ perfusion is essential to ensure organ vitality. The response to endothelin (ET)-1, a potent vasoconstrictor, is enhanced following shock, which subsequently further impairs circulation and induces organ damage. Estradiol administration post-injury attenuated ET-1 responses improving liver and intestinal perfusion [99, 100]. In addition, estradiol attenuated intestinal injury by modulating myeloperoxidase activity, TNF-α, IL-6, intracellular adhesion molecule (ICAM)-1, cytokine-induced neutrophil chemoattractant (CINC)-1, CINC-3 and macrophage inflammatory protein (MIP)-2 levels by enhancing p38MAPK and Akt pathways [101, 102]. Interestingly, treatment with the androgen antagonist flutamide exerted similar effects [103, 104].

There are currently no human studies investigating the influence of SSH on the gastrointestinal system following trauma.

#### Other body systems

Rodent studies have demonstrated gender dimorphism in responses of various organs to injury. Caruso et al. concluded that lung protection against injury was greatest during the estrus and proestrus stages of the menstrual cycle and decreased during the diestrus stage [105]. Furthermore, oestrogen and progesterone administration attenuated emphysematous and inflammatory changes in the lung as well as improved congestion and oedema following sepsis [87]. Tominaga et al. showed that oophorectomized animals displayed a 2.6-fold increase in vascular permeability following ioxaglate treatment (a contrast medium that increases vascular permeability) compared to controls and that this effect was blocked following estradiol administration [106]. These effects may be secondary to the decreased expression of keratinocyte-derived chemokines (KDC), migration inhibitory factor (MIF) and TLR-4, which in turn reduces cytokine/chemokine production and neutrophil infiltration, reducing oedema formation and subsequent organ damage [107, 108]. These protective properties of E2 are mediated via the extracellular signal-regulated protein kinase and eNOS pathways [109, 110]. In contrast, male rats displayed increased trauma-shock-induced lung injury with increased nitrite/nitrate production, hence demonstrating the potential injurious role of male SSH [45].

Research in animal models has also indicated differential neuroendocrine-immune responses between genders following neurotoxaemia. Administration of E2 abolished most of the gender dimorphic responses including hyperglycaemia, hypercorticosteronaemia and hypercytokinaemia [111]. In addition, oestrogen has been suggested to be beneficial in acute central nervous system (CNS) injuries secondary to oxidative and/or excitotoxic stress [112]. For instance, the synthesis of NO, which is known to exert harmful effects on the CNS, is induced via enhanced expression of inducible NOS following CNS injury. E2 and progesterone inhibit the expression of inducible nitric oxide synthase (iNOS) under such conditions [113, 114], and E2 administration downregulates microglial cell-mediated inflammatory responses following trauma-haemorrhage [115]. Additionally, E2 treatment may preserve the integrity of the blood-brain barrier by inhibiting matrix metalloproteinases 2 and 9 activation [116]. Interestingly, females display greater neuronal preservation than males after being subjected to injury such as ischaemia [117].

In a rabbit model of ischaemia-reperfusion injury associated with the spinal cord, testosterone exhibited neuroprotective properties by reducing caspase-3, myeloperoxidase [118] and xanthine oxidase [119] activities, as well as decreasing malondialdehyde levels and increasing catalase concentrations [120]. These results should be interpreted with caution as recent large randomized controlled trials of SSH-based interventions (Progesterone) failed to demonstrate significant benefits following severe traumatic brain injury (TBI) despite promising preclinical studies [121, 122]. Furthermore, a meta-analysis of seven randomized control trials examining progesterone influence following moderate to severe TBI concluded outcomes were not improved compared with placebo [123]. Additionally, elevated systemic levels of estradiol and testosterone following severe TBI have been associated with increased mortality and worse global outcomes for both males and females [119]. Finally, a recently published Cochrane systematic review has graded the quality of current evidence assessing progesterone’s influence of TBI as low due to substantial inconsistencies across studies, concluding present evidence do not support the proposal that progesterone reduces mortality or disability in TBI patients nor was it associated with more adverse events, and advised that more precise classification of TBI and optimisation of progesterone dosage and scheduling would benefit future trials [124].

Gender dimorphic renal responses to injury have also been reported. Male mice were more prone to renal injury. Park et al. showed that the presence of testosterone, rather than the absence of oestrogen, inhibited the activation of NOS/Akt/ERK pathway resulting in greater infiltration of leukocytes exacerbating renal cell injury and apoptosis following ischaemic insult [125]. Interestingly, low-dose testosterone demonstrated a renal protective effect following injury through modulating inflammation by enhancing intrarenal inflammatory cytokine production such as IL-10, as well as suppressing renal T cell infiltration. In contrast, high-dose testosterone displayed pro-inflammatory roles and failed to improve renal function after injury [126]. Furthermore, E2 prevented renal injury by stimulating the Akt pathway and enhancing eNOS phosphorylation [127]. Interestingly, Kasimay et al. investigated gender differences in CRF-induced oxidative multiorgan failure and found that males and oophorectomised females exhibited exaggerated systemic inflammatory responses. E2 treatment significantly improved CRF-induced systemic inflammatory outcomes in both male and female animals by modulating cytokine release and depressing tissue neutrophil infiltration [128].

### Burn injury and SSH

There are several features of the physiological response to burn injury that differ from non-burn injury and require separate consideration in relation to gender dimorphism of outcomes. Thermal injuries are associated with augmented and prolonged hypermetabolic response with resting energy expenditure up to 180% above normal values, which is more severe than other forms of trauma [129, 130]. This, along with an overwhelming immune-inflammatory response, exerts grievous effects on various body systems characterized by increased oxygen consumption, resting energy expenditure, fat and protein catabolism as well as hyperinsulinemia and enhanced peripheral insulin resistance [131–135]. All of which have a negative impact on organ/tissue function, as well as on tissue mass to the extent that it may lead to complications such as immune dysfunction, delayed wound healing and severe sepsis, as well as growth retardation [130, 136–139]. These hypermetabolic and hyperinflammatory responses have been reported to potentially endure for years post-injury [138, 140].

There is growing evidence that SSH levels are a major determinant of prognosis following burn injury. An 11-year review of data in the UK Greater Manchester region reported that the largest proportion of burn-related deaths (24.8%) was among older individuals (≥75 years in age) and that the relative risk of mortality was approximately 1.5× higher in males [141]. An analysis of the international burn injury database for England and Wales (2003–2011) concurred that patients aged 65 years or over suffered longer inhospital length of stay, as well as the highest mortality rates among all other age groups, 19.24%. Interestingly, in this analysis, mortality was generally higher in females than males over the 8-year period (1.86% vs 1.32%) and in each individual year examined [142]. This was further supported by Moore et al. who showed that risk of death in women admitted to intensive care post-thermal injury was double when compared with males, OR 2.35 [2]. Gender dimorphism in burn injury thus appears to be the opposite to other forms of injury. This is further supported by Summers et al. who concluded female gender is associated with poorer outcomes following severe thermal injury [143]. A systematic review of the literature published from 1965 till 2012 also identified female gender as a risk factor for hypertrophic scarring in patients who survived their burn injury [144].

There are a limited number of clinical studies investigating the impact of gender on outcomes following thermal injury, though animal studies offer some insight into potential mechanisms that may explain these epidemiological findings. Anathakrishnan et al. described similar responses in rats following burn injury (40% TBSA) and trauma-haemorrhage, in which both acute lung and intestinal injury were potentiated by oophorectomy and prevented by castration [45]. Wigginton et al. stated that a single intravenous dose of E2 reduced burn injury severity through regulation of the immuno-inflammatory cascade, as well as its anti-oxidant and anti-apoptotic properties [145], and other studies reported estradiol administration, following severe thermal injury, attenuated body mass loss associated with the hypermetabolic response [146]. Gregory et al. suggested that gender dimorphism relating to immune function following severe thermal injury was mediated by oestrogen and its impact on IL-6 production. This study reported that while intact females, at day 10 post-burn, exhibited three times higher levels of plasma IL-6, they also demonstrated suppression of splenocyte proliferation and delayed type hypersensitivity reactions [147]. In contrast, Gatson et al. found administration of E2 after thermal injury attenuated both brain inflammation and apoptotic signalling by down-regulating TNF-α, IL-1β and IL-6 levels within brain tissue [148]. Increasing concentrations of estradiol, through castration or treatments with E2 or anti-androgens, post-burn was also associated with reduced remote organ inflammation [149].

The data concerning the involvement of oestrogens in regulating the response to burn injury is thus mixed even in animal models, with few studies involving human patients. Comparisons of burn injury outcomes in pre- and post-menopausal women or those on HRT would be beneficial in this respect.

### Therapeutic potential of SSH

#### Anabolic androgenic steroids (AAS)

Oxandrolone is an AAS that is derived from testosterone and has a high anabolic:androgenic ratio (10:1) [150]. Oxandrolone has been shown to improve prognosis of various catabolic conditions including severe burns and trauma [151]. It is the only AAS approved by the FDA for weight restitution following extensive surgery and severe trauma.

To date, there has been one multicenter prospective randomized double-blind trial investigating the effects of oxandrolone in adult patients with severe burns. The authors reported significantly shorter lengths of inhospital stay in the oxandrolone group compared to placebo, and this difference was strengthened when deaths were excluded and hospital stay indexed to burn size [152]. A recent meta-analysis of 15 randomized controlled trials reported that oxandrolone use was associated with shorter inhospital length of stay by 3 days, reduced donor site healing time by 4.4 days, and reduced time between surgical procedures by 0.7 days, as well as reduced weight loss by 5 kg and nitrogen loss by 8.19 g/day. Moreover, oxandrolone use in the rehabilitation phase was associated with reduced weight loss by 0.86 kg/week and lean body mass by 5% as well as gaining 3.99% and 10.78% lean body mass following severe thermal injury by 6 and 12 months respectively [153]. Interestingly, oxandrolone and propranolol (β-blocker used in burns for its anti-catabolic effects) attenuated burn-induced growth arrest in paediatric patients following thermal injury by shortening its duration by 84 days and increased growth rate by 1.7 cm per year [154]. The use of oxandrolone in paediatric burn patients up to 2 years is associated with greater improvements in bone mineral content, bone mineral density and height velocity [155].

#### DHEA/DHEAS

DHEA, a major steroid hormone circulating in plasma, is produced in response to stress and is an intermediate that can be metabolized to both testosterone and oestrogen. It has been reported to exhibit predominantly oestrogenic effects in the male androgenic milieu [156]. In view of the immuno-enhancing properties of oestrogen, studies have investigated the effect of DHEA in animal models of trauma-haemorrhage and sepsis. Angele et al. demonstrated that administration of DHEA attenuated depression of splenic and peritoneal macrophage function post-injury and improved mortality rates from subsequent sepsis in a rodent model [157]. Furthermore, DHEA, in post-trauma-haemorrhage, restored splenocyte functions by directly stimulating T cell functions and preventing increases in serum corticosterone [158]. Interestingly, DHEA has been shown to antagonize the immunosuppressive effects of glucocorticoids such as dexamethasone on lymphocyte proliferation [159], and the sulphated form of DHEA, DHEAS, has been shown to potentiate neutrophil function via direct activation of neutrophil nicotinamide adenine dinucleotide phosphat (NADPH) oxidase and reactive oxygen species (ROS) generation [160].

There are no human trials of DHEA intervention in trauma, and this androgenic hormone has mainly been used in trials for Addison’s disease and some chronic inflammatory conditions including rheumatoid arthritis. As the HPA axis is disrupted after trauma, we suggest that supplementation with DHEA may offer a novel, safe and inexpensive route in improving a range of outcomes after injury.

#### Androgen receptor antagonists

Several animal studies have indicated that testosterone depletion exerts numerous beneficial effects prior to any systemic insult. Administration of flutamide following trauma-haemorrhage and resuscitation normalized depressed splenic and peritoneal macrophage cytokine release [161]. Angele et al. showed that flutamide administration for three consecutive days not only restored diminished immuno-inflammatory responses but also decreased mortality rates associated with subsequent septic challenge [162]. Lin et al. evaluated the use of flutamide in animal models of heatstroke, reporting that flutamide attenuated hypothermia; decreased the number of apoptotic cells within the hypothalamus, spleen, liver and kidney; diminished the plasma index of toxic oxidized radicals such as nitric oxide metabolites; attenuated systemic inflammatory responses including TNF-α and IL-6 release; and reduced the infiltration of neutrophils into the lungs. All of which contributed to significantly improved mortality rates [163]. Furthermore, flutamide is frequently used in the clinical management of testicular cancer over prolonged periods without major adverse effects. Therefore, short-term use can be considered safe and feasible. Again, there are currently no human studies investigating administration of androgen antagonists following trauma or burn injury.

## Conclusions

**Fig. 1:**
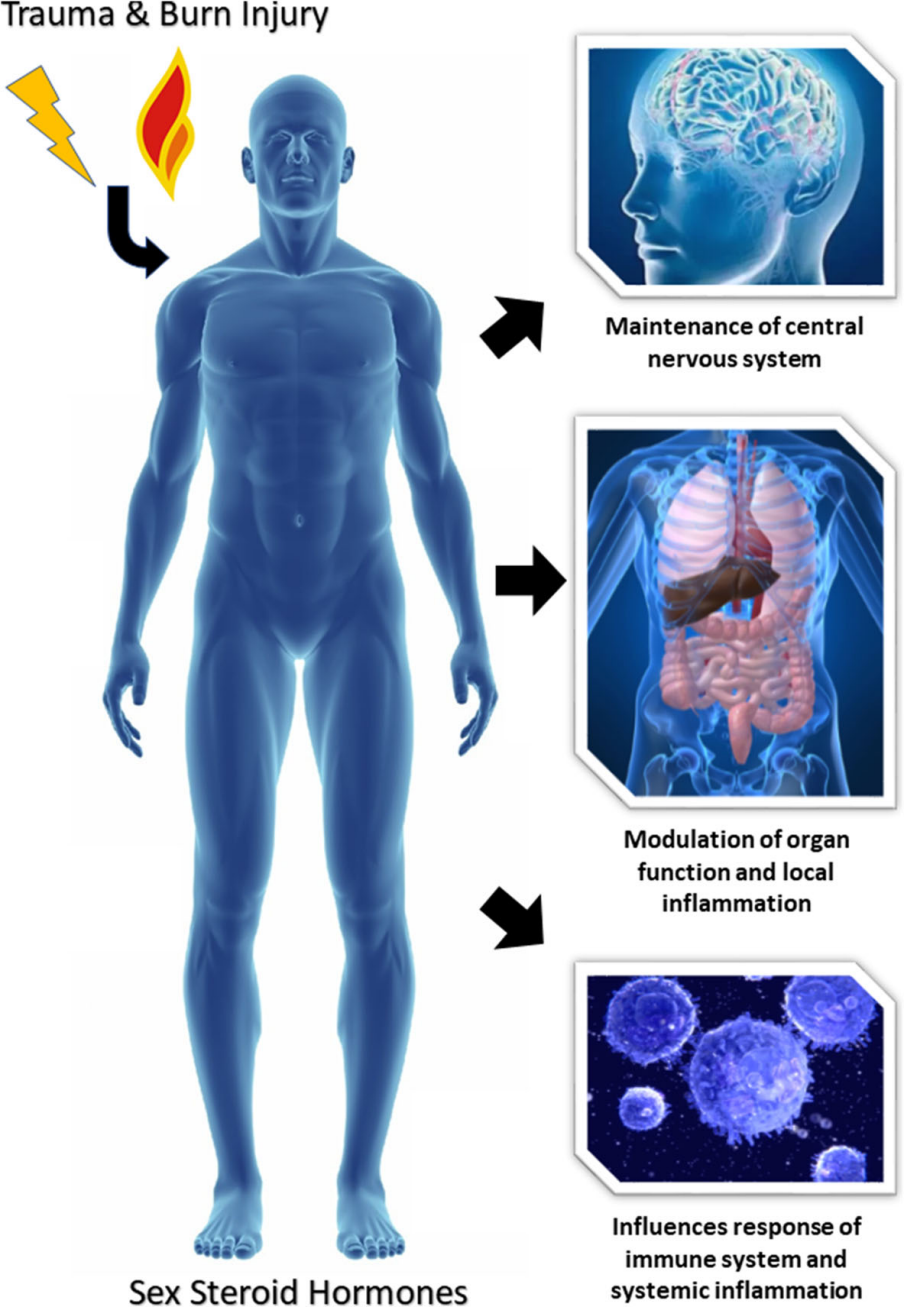
The influence of SSH on the human body response following injury

The literature contains evidence of gender dimorphism in response to injury, with outcomes better in females than males for most injury types, one possible exception being burn injury. SSH have demonstrated potential to support homeostatic measures following injury by modulating a wide range of processes including inflammation, immune response and organ function (Figure 1). However, at present, most of these data are derived from in vitro or animal-based studies and conclusive clinical trials of interventions with SSH are lacking. Further investigations are merited to ascertain the role of specific SSH in post-injury pathology as their therapeutic potential may prove invaluable in reducing patient morbidity and mortality in the clinical setting.
